# Key Drivers to Implement an Evidence-based Tobacco Control Programme in Schools of India: A Mixed-Methods Study

**DOI:** 10.31557/APJCP.2021.22.2.419

**Published:** 2021-02

**Authors:** Akash Pradhan, Kunal Oswal, Keyuri Adhikari, Ajita Singh, Rishav Kanodia, Lakshman Sethuraman, Ramachandran Venkataramanan, Glorian Sorensen, Eve Nagler, Mangesh Pednekar, Prakash Gupta, Arnie Purushotham

**Affiliations:** *Program Manager, Public Health, Cancer Care Program, Tata Trusts, India.*

**Keywords:** Tobacco control, prevention, school, teachers, educational institutions

## Abstract

**Background:**

Adolescence is an influential stage in students’ lives when lifelong behaviours such as tobacco use are formed. During these years, school teachers are important role models for tobacco control among students. A study was conducted among school personnel and administrators to understand the key drivers for implementing an evidence-based school tobacco control program.

**Methodology:**

A cross-sectional, mixed-method study was conducted in five districts of Assam, India. The quantitative study was conducted among 565 school personnel across 40 Government-aided schools. Data was collected by means of an anonymous, self-administered questionnaire. Qualitative data was generated from 15 focus group discussions (FGDs) among 146 participants - District Program Officers, Block Education Officers, Cluster Coordinators, Headmasters and Teachers.

**Results:**

While the prevalence of smoked tobacco was low (3%), the use of smokeless tobacco was higher (40%), and the prevalence of use of areca nut without tobacco (65%) was still higher among school personnel. They were aware of the school policies prohibiting the use of tobacco among students within or outside school buildings or during school-sponsored activities (81%); they had rather limited knowledge about policy for themselves (58%). There was lack of access to training materials about prevention of tobacco use among youth. The FGDs amongst school personnel resulted in several constructive suggestions on tobacco control in schools mainly in training school teachers, monitoring the program and incentives for execution of the program. However, there was a reluctance to implement a smokeless tobacco control programme since many were current users of smokeless tobacco and areca nut.

**Conclusion:**

Tobacco control policies as well as training school personnel in schools need to improve and further measures must be taken to prohibit use of areca nut, which contains carcinogens. The existing system of the education department can be utilised to implement tobacco control programmes effectively.

## Introduction

Tobacco is a major risk factor for cancer and other non-communicable diseases and is the single largest preventable cause of deaths worldwide (WHO, 2020). Globally, approximately 8 million people die each year from diseases due to tobacco consumption (WHO, 2020). If effective steps are not taken to control, by 2030 tobacco attributable deaths is expected to rise to more than 10 million per year, with 70% of them occurring in low and middle income countries (LMIC) (Mackay and Eriksen, 2002). 

India is the second largest producer and consumer of tobacco, 28.6% of adults aged 15 years and above (266.8 million people) use some form of tobacco (WHO, 2016; Businesses Wire, 2019). In addition to the smoked forms that include cigarettes, bidis and cigars, a plethora of smokeless forms of consumption exist in India (Reddy and Gupta, 2004). Some commonly used smokeless tobacco products are betel quid (made from betel leaf, areca nut, slaked lime, catechu and other flavouring agents), khaini (sun-dried or fermented coarsely cut tobacco leaves mixed with slaked lime), mawa (mixture of thin shavings of areca nut with some tobacco flakes and slaked lime), mishri (roasted and powdered tobacco), tamool (mixture of tobacco, areca nut, noura, slaked lime, catechu, tamool leaf, powdered khat and other flavoring ingredients.), gutkha (made from areca nut, slaked lime, catechu and sun-dried, roasted, finely chopped tobacco with flavorings and sweeteners) and many others (WHO, 2021; Joshi et al., 2011). Use of both smoking and smokeless tobacco is associated with increased risk of chronic and terminal diseases. (Hettiarachchi et al., 2020). 

Areca nut, which is known to cause oral cancer, is used by over 600 million people across the world and is the fourth most widely used addictive substance with India being the largest consumer (WHO, 2004; Gupta et al., 2018; Warnakulasuriya and Peters, 2002). It is consumed in South East Asia, China and the Pacific Islands. In countries like the Gulf, North America and Europe, immigrants of Asian and Pacific Countries consume areca nut (Warnakulasuriya and Peters, 2002). Approximately, 90% of oral cancers are due to chewing habits including with areca nut and smoking (Jeng et al., 2001; Jiang et al., 2019).

Assam is the third largest tobacco producer in India and has one of the highest rates of tobacco consumption among all the other states with 48.2% of all adults who either smoke tobacco and/or chew tobacco. The mean age of initiation of tobacco use in Assam is 18.5 years (WHO, 2016). As a part of Global Tobacco Surveillance System (GTSS) initiative, a Global Youth Tobacco Survey (GYTS) conducted in Assam, prevalence of ever tobacco use among school children was 40% and current tobacco use was 36% (Sinha et al., 2003).

It is important to understand the pattern and behaviour of school personnel and whether they can act as role models for tobacco control as well as have an impact on school children. As adolescence (10 -19 years) is an influential stage in students’ lives, lifelong behaviours such as tobacco use are formed at this stage (Poulsen et al., 2003). During these years, school teachers are important role models. If, however, teachers themselves use tobacco, especially in the presence of students on school premises, that gives a message to children that there is nothing wrong in using tobacco (Poulsen et al., 2003). In the Global School Personnel Survey, the prevalence of daily or occasional smokeless tobacco use among school personnel in Assam was 45.4% and smoking, 51.7% (Sinha et al., 2003).

In order to plan tobacco control interventions in schools, it is important to understand the extent and type of tobacco use among school personnel, their attitudes towards tobacco control, and the existence of school health policies about tobacco.

The aim of the study was to: (1) Obtain information on current tobacco use among school personnel in Assam. (2) Evaluate the current state of implementation and enforcement of tobacco control policies in schools. (3) Understand the level of enforcement of tobacco control policies in the school. (4) Understand the knowledge and attitudes of school personnel towards tobacco control policies. (5) Understand the existing training and operational systems in place at the district, block, cluster and institution levels in the Education Department of Assam. 

## Materials and Methods

**Figure 1:**
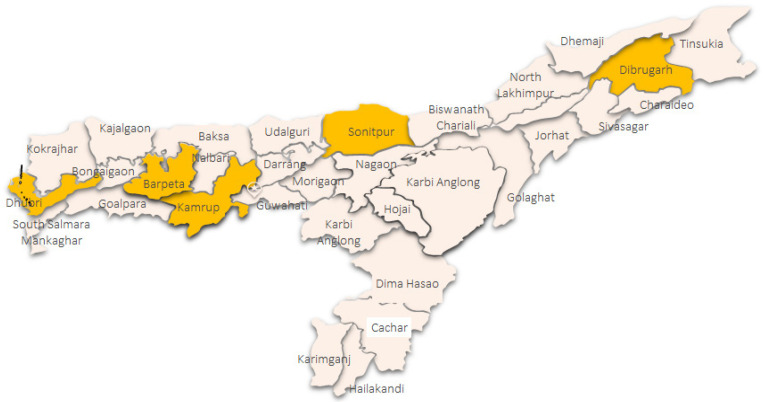
Assam Administrative District Map and District where the Data was Collected (highlighted in yellow)

The study involved data collection through both quantitative methods: (i) self-administered cross-sectional survey of all school personnel (headmasters, teachers, school health personnel staff, clerical and administrators) of randomly selected schools; and qualitative methods (ii) focus groups with representative principals, teachers and cluster coordinators (administrative head of the cluster) in the selected 5 districts. Considering the diversity of settings within Assam, to obtain representation across the state, two districts from upper Assam (Dibrugarh, Sonitpur) and three from lower Assam (Barpeta, Dhubri, Kamrup- Rural) were chosen (Figure 1) (Department of Science and Technology, 2020). 

This study sample were from government and government-aided schools representing rural and urban areas of Assam. Lists of all (2103) eligible schools in the stated five districts of Assam having grades 8–10 were obtained. Schools were identified through their districts (highest school administrative unit), and clusters (lowest school administrative unit), wherein a cluster was a sub-unit of the block and the block was a sub-unit of school districts in Assam. Out of total 2103 eligible schools, 40 schools were sampled by probability proportional to school personnel size. Assam, India. 

In order to recruit schools for the self-administered surveys, the Global School Personnel Survey (GSPS) methodology was used (CDC, 2020). The probability of schools selected was proportional to the number of students enrolled in the specified grades. At the second sampling stage, classes within the selected schools were randomly selected. The GSPS sample design produces representative, independent, cross-sectional estimates for each site. 

The independent state samples were designed to be representative of students and school personnel in each district. For each survey, a weighting factor was applied to each school and teacher record to adjust for the probability of selection at the school level and for non-response at the school and school personnel level. Data was collected by means of an anonymous, self-administered questionnaire. The study questionnaire was adopted from the GSPS and translated into the local language, Assamese (GTSSCG, 2006). A questionnaire consisting of 28 questions, which assessed individual characteristics of the participants (i.e. demographics, tobacco use history, self-efficacy, attitude towards and motivation to adopt tobacco control programs and policies). 

Principals, teachers and cluster coordinators were invited to attend focus group discussions (FGDs) in each chosen district. FGDs were conducted at a centralized location within each district and were conducted by trained moderators. FGDs were conducted in local languages and Hindi in the respective districts, transcribed, and translated into English. Fifteen focus groups were conducted in the five districts, one with the cluster coordinators, one with the headmasters and one with the teachers. About 8-10 participants were included in each FGD. An independent team consisting of one moderator and one note taker were involved in each district. We also conducted Key Informant Interviews with the District Program Officers (administrative head of the district) and Block Education Officers (administrative head of the block) within all five districts. For the qualitative data, an open-ended moderator guide was developed in which questions to be asked were specified. Questions explored details of the educational infrastructure for training and support, perceptions and reactions to tobacco cessation program; applicability of the program’s materials and approaches within their settings; barriers and facilitators of adopting the tobacco cessation program; resources available to implement the program in schools; adaptations needed for the program; and optimal ways to promote it to school principals. Mock sessions during trainings were conducted for the field investigators in Assamese for both the quantitative and qualitative studies. The Institutional Ethics Committee of Healis – Sekhsaria Institute for Public Health, Mumbai, approved study methods and materials. 

*Data Analysis*

*Quantitative*

Analysis of the survey data involved descriptive statistics of the school personnel’s demographics, tobacco use history, self-efficacy, attitude towards and motivation to adopt tobacco control programs and policies. 

*Qualitative*

Focus group discussions were analysed by the authors by conducting concentrated reviews of the data, reflecting upon them, and formulating independent interpretations before collectively agreeing upon the major ideas. Initial content analysis was done, and broad recurring themes were identified. 

## Results

*Quantitative Study*

All 40 sampled schools participated in the study. A survey participation response rate of 84% (565/671 participants) was obtained. The demographic characteristics of the participants in Table 1, accounting for 65% male and 35 % female participants. Majority (95%) were in the age group of 30-59 years and 79% of them were teachers, 7% headmasters.

*Tobacco use prevalence among school personnel (* Table 2 *)*

Out of 565 respondents, 65 % were males, 19 (3%) were current smokers, 125 (22%) were past smokers and 421 (75%) had never smoked. About 98 % of respondents had not smoked on school premises in the last year. Of the total respondents, 223 (40%) currently used smokeless tobacco, 84 (15%) had previously used smokeless tobacco and 250 (45%) had never used smokeless tobacco products. In all, a minority of 40 (7%) participants had used smokeless tobacco on school premises in the last year. The prevalence of use of areca nut was high. Out of the total respondents, 365 (65%) used areca nut without tobacco and 82 (15%) used areca nut with tobacco. In all, 187 (33%) used areca nut without tobacco and 56 (10%) used areca nut with tobacco on school premises in the last year. 

*School policies prohibiting use of tobacco (* Table 3 *)*

In total, 503 (89%) respondents said their school had a policy or rule specifically prohibiting tobacco use among students inside school buildings and 171 (30%) said there was such a policy or rule for school personnel. In all, 290 (51%) respondents said their schools had a policy or rule specifically prohibiting tobacco use among students outside school buildings while 201 (36%) said there was such a policy or rule for school personnel. 

About 460 (81%) respondents also said their schools had a policy or rule specifically prohibiting tobacco use among students at school sponsored activities while 330 (58%) said there was such a policy or rule for school personnel. Finally, only 118 (21%) respondents had access to teaching and learning materials about tobacco use and how to prevent its use among youth and only 84 (15%) had received training to prevent tobacco use among youth.

*Attitudes among school personnel towards tobacco control (* Table 4 *)*

School personnel were in favour of incorporating policies or rules prohibiting tobacco use among students (95%) and school personnel (95%) on the school premises. About 400 (71%) said that they were concerned about tobacco use among youth in the community. As many as 528 (94%) respondents agreed that tobacco use by teachers influences students. In all, 548 (97%) respondents had advised students at least once to stop using tobacco and 517 (92%) respondents said teachers need specific training to be able to teach students on how to avoid or stop using tobacco.

Of the respondents, 494 (88%) thought that tobacco companies deliberately encourage youth to use tobacco and 428 (76%) were in favour of banning smoking in public places with 437 (77%) respondents agreeing that there is a need to increase prices of tobacco products.

*Knowledge among school personnel on tobacco use (* Table 5 *)*

In all, 425 (76%) respondents stated that tobacco is addictive, 467 (83%) were aware that tobacco causes lung cancer and 421 (75%) were aware that tobacco causes heart disease. 

*Qualitative Study (* Table 6 *)*

*Learnings from the Focus Group Discussions*

The major learning themes that emerged from the focus group discussions (FGDs) were mainly on training of school teachers, headmasters and cluster coordinators, regular monitoring of the tobacco control program and some form of incentivization might help in execution of the program. 

*Suggestions for implementation of tobacco control programs*

*Training*

**Figure 2:**
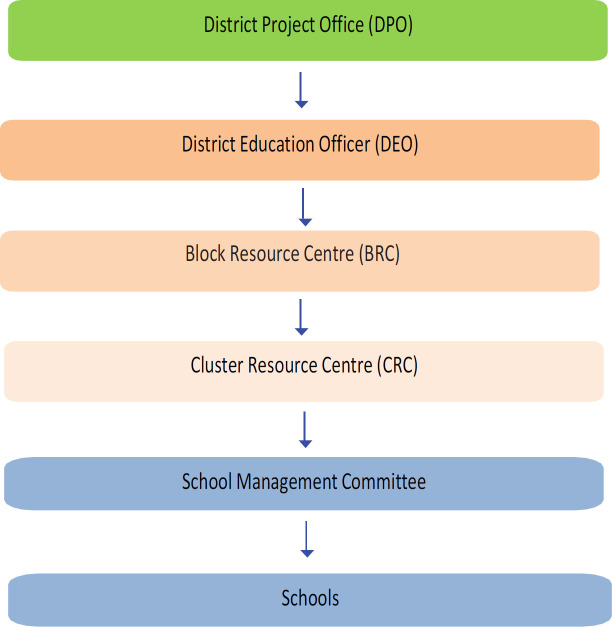
Administrative Structure of the Education System in Assam

The education structure enabled us to understand the process of working from state to district level authorities (Figure 2) (Samagra Siksha Abhiyan, 2020). The FGDs suggested that about one hour of any one of the monthly cluster-level meetings with headmasters and teachers could be utilised for tobacco control training (training of school teachers in tobacco-free school guidelines, harms of tobacco, tobacco control laws). Head teachers could implement the same with the help of teachers and students through the School Management Committee (committee mainly comprised of headmaster, supervisor, teachers and trustees).

Health and Education goes hand in hand. Whenever any health related program is introduced, it is usually for the students and teachers have to implement it in schools… This is the first time that a program has come in for us teachers and for our health… Everyone knows the harms of tobacco and still uses it. No one has ever conducted an awareness program for us... We will definitely provide our support…’

*Cluster Coordinator, Kamrup Rural Monitoring*

Cluster coordinators suggested that the program could be monitored and tracked during their monthly visits, and followed up through WhatsApp Groups. Telephone calls with the cluster coordinators and block officers could also be used to resolve queries. It was suggested by the cluster coordinators that specific modules could be developed as guidelines for implementation, along with specific measuring tools and progress parameters. 

‘*Creating awareness is okay. However, you should not target only teachers. We are not the only one who are using tobacco. Children are exposed to it at their homes as well; we should focus on that also. Government should ban all forms of tobacco, if it is so harmful. This will prevent everyone from using it*’ - A Headmaster, Dhubri

*Incentivisation*

Regular support from higher authorities in the district or the state level along with an incentive and accountability in system for effective program implementation could be a motivating factor for the school personnel and the students. 

‘*This program looks good; it will definitely create a much needed impact! We have many officials who use tobacco. An incentive mechanism to be in place to deter tobacco use with school personnel. But we should also pass it on to the students… as creating a new generation that is tobacco free is more important.*’ - District Program Officer, Barpeta

However, it was observed that the majority of the focus groups participants were reluctant in implementing smokeless tobacco control programs, as some were current users. Further, it was also highlighted that in few of the schools the human resource was understaffed and infrastructure was limited (projector, training material), which could impact an effective program implementation. 

**Table 1:** Sociodemographic Details of the Participants

Demographic characteristics of distribution	Frequency (N=565)	Percentage
Geographical Distribution of respondents		
Barpeta	178	32%
Dhubri	108	19%
Dibrugarh	99	18%
Kamrup –R	138	24%
Sonitpur	42	7%
Gender		
Female	196	35%
Male	368	65%
Did not respond	1	0%
Age of the Participants		
Less than 19 Years	2	0%
20 to 29 Years	18	3%
30 to 39 Years	116	21%
40 to 49 Years	159	28%
50 to 59 Years	266	47%
60 Years older	3	1%
Did not respond	1	0%
Position of Participant in the school		
Administrator/Headmaster	39	7%
Teacher	443	78%
School Health Service Personnel	10	2%
Clerical Staff	29	5%
Other type of school Personnel	41	7%
Did not respond	3	1%

**Table 2:** Tobacco Use Pattern among the School Personnel in the Study

Tobacco use prevalence among school personnel	Frequency (N=565)	Percentage
Current status of smoking of smoke cigarettes, bidis etc		
Currently smoking	19	3%
Past smoker	125	22%
Never smoked	421	75%
Have you ever smoked cigarettes on school premises/property during the past year?		
Yes	8	1%
No	548	97%
Did not respond	9	2%
Current status of smokeless tobacco use		
Currently use	223	40%
Past use	84	15%
Never used	250	44%
Did not respond	8	1%
Have you ever used chewing or applying tobacco or snuff, on school premises/property during the past year?		
Yes	40	7%
No	522	92%
Did not respond	3	1%
Have you ever used areca nut or tamool without tobacco?		
Yes	365	65%
No	197	35%
Did not respond	3	0%
Have you ever used areca nut or tamool without tobacco on school premises/property during the past year?		
Yes	187	33%
No	376	67%
Did not respond	2	0%

**Table 3:** Knowledge about School Policies Prohibiting Use of Tobacco

School policies prohibiting use of tobacco	Frequency (N=565)	Percentage
Does your school have a policy or rule specifically prohibiting tobacco use among students inside school buildings?		
Yes	503	89%
No	48	8%
I don’t know	11	2%
Did not respond	3	1%
Does your school have a policy or rule specifically prohibiting tobacco use among school personnel inside school buildings?		
Yes	171	30%
No	364	64%
I don’t know	26	5%
Did not respond	4	1%
Does your school have a policy or rule specifically prohibiting tobacco use among students outside school buildings, but on school premises/property?		
Yes	290	51%
No	214	38%
I don’t know	56	10%
Did not respond	5	1%
Does your school have a policy or rule specifically prohibiting tobacco use among school personnel outside school buildings, but on school premises/property?		
Yes	201	36%
No	282	50%
I don’t know	76	13%
Did not respond	6	1%
Does your school have a policy or rule specifically prohibiting tobacco use among students at school sponsored activities wherever they occur?		
Yes	460	81%
No	67	12%
I don’t know	35	6%
Did not respond	3	1%
Does your school have a policy or rule specifically prohibiting tobacco use among school personnel at school sponsored activities wherever they occur?		
Yes	330	58%
No	187	33%
I don’t know	43	8%
Did not respond	5	1%
Do you have access to teaching and learning materials about tobacco use and how to prevent its use among youth?		
Yes	118	21%
No	442	78%
Did not respond	5	1%
Have you ever received training to prevent tobacco use among youth?		
Yes	84	15%
No	478	84%
Did not respond	3	1%

**Table 4:** Attitudes among School Personnel towards Tobacco Control

Attitudes among school personnel towards tobacco control	Frequency (N=565)	Percentage
Do you think schools should have a policy or rule specifically prohibiting tobacco use among students on school premises/property?		
Yes	538	95%
No	24	4%
Did not respond	3	1%
Do you think schools should have a policy or rule specifically prohibiting tobacco use among school personnel on school premises/property?		
Yes	540	95%
No	22	4%
Did not respond	3	1%
How concerned are you about tobacco use among youth in your community?		
Very	400	71%
Somewhat	151	27%
Not	13	2%
Did not respond	1	0%
Do you think teacher tobacco use influences youth tobacco use?		
Yes	528	94%
No	32	5%
Did not respond	5	1%
Have you ever advised a student to stop using tobacco?		
Yes	548	97%
No	17	3%
Do you think teachers need specific training to be able to teach students how to avoid or stop using tobacco?		
Yes	517	92%
No	46	8%
Did not respond	2	0%
Do you think the tobacco industry deliberately encourages youth to use tobacco?		
Yes	494	88%
No	69	12%
Did not respond	2	0%
Are you in favor of banning smoking in public places?		
Yes	428	76%
No	135	24%
Did not respond	2	0%
Do you think the price of tobacco products should be increased?		
Yes	437	77%
No	121	21%
Did not respond	7	1%

**Table 5:** Knowledge among School Personnel on Tobacco Use

Knowledge among school personnel on tobacco use	Frequency (N=565)	Percentage
Is tobacco use addictive?		
Yes	425	76%
No	31	5%
I don’t know	107	19%
Did not respond	2	0%
Does tobacco use cause lung cancer?		
Yes	467	83%
No	9	2%
I don’t Know	87	15%
Did not respond	2	0%
Does tobacco use cause heart disease?		
Yes	421	75%
No	19	3%
I don’t know	124	22%
Did not respond	1	0%

**Table 6:** District-Wise Distribution of Participants for Qualitative Study

District-wise list of participants for the quantitative study						
Districts	District Program Officers	Block-level officers	Cluster Coordinators	Headmasters	Teachers	Total
Barpeta	-	8	9	3	4	24
Dhubri	5	6	8	11	-	30
Dibrugarh	-	-	12	12	-	24
Kamrup – R	-	11	7	7	11	36
Sonitpur	-	4	11	17	-	32
					Total	146

## Discussion

While the prevalence of smoked tobacco was low (3%) and use of smokeless tobacco higher (9%), the prevalence of use of areca nut without tobacco (65%) and with tobacco (15%) was very high among school personnel. The majority of the school personnel (81%) were fully aware of the policy for prohibiting the use of tobacco either within or outside the school premises for school children but only 58% of the school teachers were fully aware of the school policies prohibiting the use of tobacco either within or outside school buildings or during school- activities for themselves. There was a lack of access to training materials about prevention of tobacco use among youth. There was overwhelming support for prohibiting use of tobacco on school premises and receiving training to help students to cease initiation or quit tobacco use. The focus group discussion amongst school personnel resulted in several constructive suggestions on tobacco control in schools. However, there was a reluctance to implement a smokeless tobacco control programme since some were current users of smokeless tobacco and areca nut.

About 3% of school personnel in this study reported that they were current smokers of cigarettes, and bidis, comparable to a study from Botswana, which showed that the prevalence of smoking among teachers was 3% (Erick, 2013). This prevalence of tobacco use is relatively low compared with results from other studies conducted amongst school teachers in Odisha (smoked tobacco - 28%; smokeless tobacco 68%) and Bihar (smoked tobacco - 43%; smokeless tobacco 57%) (Sinha and Gupta., 2004; Sinha et al., 2004). While, in a recent survey conducted in Sri Lanka among 212 nursing students showed that 73.1% knew that “areca nut is an addictive substance”, 76% respondents in our study stated that tobacco is addictive (Hettiarachchi, Jayasooriya, Amarasinghe, et al, 2020).

The use of smokeless tobacco is widely prevalent in South East Asia. National survey findings conducted in 3 countries of Southeast Asia Regional Office (SEARO) WHO, the prevalence rate of use of smokeless tobacco in men aged between 15 and 49 years was 23% (Bangladesh), 33% (India) and 32% (Nepal) (Sinha et al., 2015). Despite a ban on cultivation, manufacture, distribution and sale of tobacco since 2004 in Bhutan, a survey conducted in 2014 of 2820 adults aged 18 – 69 years showed a prevalence of 7.4% smoked tobacco use, 19.7% smokeless tobacco use and 2.3% dual use. There was a higher prevalence in males, younger age and alcohol users (Gurung et al., 2014). A comparison between the use of smokeless tobacco in Sweden where it is legal to sell snus and 17 other countries in Europe where sale of smokeless tobacco is restricted, in 1000 individuals >15 years this showed a prevalence of 20.7% in men in Sweden compared with 1.1% in the remaining countries combined (Leon et al., 2016).

Areca nut is consumed mainly in South East Asian countries (India, Sri Lanka, Bangladesh, Myanmar, Maldives, Pakistan and Taiwan). It is also reported from the Association of Southeast Asian Nations (ASEAN) countries like Thailand, Malaysia, Indonesia, Cambodia, Vietnam, Laos, and China with the immigrants from these countries using it globally in North America, Europe and Gulf countries (WHO, 2004). The usage of areca nut in Southeast Asia is interwoven with the traditional customs, rituals, local art and craft, and religious practices. An estimated 10 -20 % of world population use areca nut in some form (WHO, 2004). Data from studies show a wide point prevalence of areca nut use (1- 54%). Strickland reviewed in detail the identity of historical and contemporary users of the areca nut to determine the reasons given by users for consuming areca nut, drawing upon historical, ethnographic and experimental sources of evidence for the effects, which users derive from it. The pattern of use showed that that more women than men consume areca nut with early onset of use continuing more frequently with increasing age whereas in the present study more men than women consume areca nut (Strickland, 2002).

The relatively higher prevalence of areca nut use in this study can be attributed to the age-old cultural and traditional practice of the people of Assam. The capital of Assam, Guwahati, derives its name from the Assamese words “*Guva*” derived from the Sanskrit word Guvaka, meaning areca nut and its plant and “*Hati*” meaning rows, the row of areca nut trees (Sharma, 1978). Most people in this region chew betel nut with betel leaf. Many like the combination of slaked lime and raw tobacco along with the betel nut and leaf, which is carcinogenic and contributes to the high incidence of oral cancers in the region (Adhikari et al., 2016). The influence of tradition and culture should not be underestimated. In Assam, for instance, a wedding is incomplete without betel nut. From inviting one’s guests and throughout the wedding celebration, betel nut forms an important component. In important festivals like Bhogali Bihu, which is a harvest festival, betel nut along with other items like rice cakes or pithas, are burned as an offering to the God of Fire (NDTV, 2017).

In both the qualitative and quantitative studies, the use of areca nut among school personnel was found to be very common. The tobacco control practices in the school should also include prohibition of areca nut with or without tobacco due to the ill effects of its use and its direct link to oral cancers. 

The huge gap in policies among schools prohibiting tobacco use on school premises and at school-sponsored activities suggests that there is room for significant improvement. These findings also indicate that teachers are supportive of tobacco control policies in schools. The Government of India released the “Guidelines for Tobacco-Free Schools/Educational Institutions” in 2019 and “Step by Step Guidelines for implementation of Section 6b of the Act and Rules” in 2017 (Ministry of Health and Family Welfare, 2019; Ministry of Health and Family Welfare, 2013). The objective of these guidelines was to provide fresh momentum to the implementation of tobacco control initiatives among adolescents and young adults. These guidelines will play a pivotal role in implementing tobacco control measures in educational institutions, which will influence positive change in and around them, potentially leading to higher quit rates and lower rates of initiation of use of tobacco among the students. 

In conclusion, tobacco control policies in schools need to improve and further measures must be taken to prohibit use of areca nut. The existing system of the education department can be utilised to implement tobacco control programmes effectively. An intervention model designed to influence schools and departments of education can be leveraged in order to have a wider impact on patterns of tobacco and areca nut use among teachers, and ultimately to shape community and student norms around use.
